# Ophthalmology

**Published:** 1906-09

**Authors:** Cyril H. Walker


### OPHTHALMOLOGY.

The **Giant Magnet for the removal of pieces of steel from the eyeball,** though invented by Haab some fourteen years ago, has only come into general use during the last five or six years. One of these instruments was fitted up at the Bristol Eye Hospital about two years ago, and is worked with the Corporation 250 volt power (continuous) current. Numerous cases of successful use of the giant magnet are on record. Some details of its management are given in a paper by Wharton.[1] The following points are of importance:—

1. When the position of the fragment is not known it should, if possible, be ascertained by means of an X-ray photograph. 2. It is important that the magnet should be tried as soon as possible, before the fragment has become embedded in plastic exudation. 3. The prognosis, where the fragment is in, or in front of, the lens, is good: where it has penetrated the lens and lies in the vitreous it is very bad. In such cases it might be possible to make an incision in the sclerotic near the foreign body, rather than to attempt to remove it through the injured lens. Haab describes the process of coaxing the fragment from the posterior surface of the lens round its equator and through the pupil into the anterior chamber; but this must be very rarely possible unless (which is seldom the case) the movement of the fragment can be seen and followed. 4. The possibility of the fragment, though, steel or iron, being non-magnetic, must be borne in mind. Snell, in a paper read before the Ophthalmological Society,[2] draws attention to the fact that some steel alloys are nearly, if not quite, non-magnetic. Manganese steel (12 per cent. manganese), and nickel steel, with even less than 1 per cent. manganese, are practically non-magnetic.

**Extraction of Cataract in the Capsule.**—Until about fifteen months ago little was heard of this operation in Europe and America, though it was known to be practised occasionally by oculists in India. Towards the close of last year Major Henry Smith, a civil surgeon in the Punjaub, published details of the operation.[3] His experience extends over 11,000 cataract extractions—2,000 performed in the orthodox method of scratching the capsule and leaving it behind, and 9,000 extractions in the capsule. He concludes that cataracts in juveniles, atrophic cataracts, and gelatinous cataracts are unsuitable for extraction in the capsule. He gives the following description of the operation:—"I make a liberal-sized upper incision, inserting the knife at the sclero-corneal junction, just as deep as anatomy and experience teach us will avoid wounding the dangerous area, and cut out in the cornea with a sweep

[1] *Ophth. Rev.*, December, 1905. [2] *Brit. M. J.*, 1906, i. 1465.

[3] *Arch. Ophthal.*, 1905, xxxiv. 601.

half-way between a normal pupil and the sclero-corneal junction. I then take out the speculum, and my assistant hooks up the upper eyelid on an ordinary large-sized strabismus hook, and draws down the lower lid by placing the ball of his thumb on the skin of the face close to the lower eyelid. . . . It is important that he should lift the upper lid well up and retain the lower one so well down that the orbicularis muscle cannot be brought into action by the patient until the operation is finished. . . . I then place the curve of a strabismus hook over the cornea about the junction of the lower with the middle third of the lens, and a spoon just above the upper lip of the wound. I press the strabismus hook down neither towards the wound nor from it, and do not alter its position until the lens is nearly out, all the time making slow, steady, and uninterrupted pressure and counter-pressure. When the lens is more than half-way out, I, while keeping up the tension with the spoon in its original position, shift the strabismus hook forward and gently tilt the lens by getting the edge of it in the concavity of the strabismus hook. . . . The lens in its capsule being out, the eyelids are let go and bandaged up with the usual antiseptic pad. If a trace of vitreous has escaped it is snipped off with scissors, and if the iris prolapses it is replaced before the eyelids are let go. . . . There is no advantage in the preliminary instillation of atropine. . . . The operator who attempts to extract the lens in its capsule, either with or without an iridectomy, as rapidly as is done when the capsule is scratched, will have disastrous results. By over-rapid expression the capsule will burst. I find that the whole operation takes from two to three minutes. . . . The operation I perform with an iridectomy differs in no way from the above, except in the iridectomy. The corneal wound I make is very little larger than is necessary for the safe extraction of a cataract by the ordinary method."

One of the difficulties of the operation is the premature rupture of the capsule when the lens is partly out. This occurs in about 8 per cent. of Smith's cases. The prolapsed capsule and retained lens matter should in such cases be dragged out with forceps. Escape of vitreous, which occurred in more than 6 per cent. of his cases, he does not regard as serious. It will be noticed that after the corneal section is made no instrument need be introduced into the eye. In this respect the operation is a great improvement on Pagenstecher's extraction of the lens in its capsule by means of a scoop.

Maynard, of Calcutta, gives his experience of 175 cases of "Smith's" operation.[1] He prefers to call it expression rather than extraction of cataract. He makes the ordinary sclero-corneal section with conjunctival flap, thus securing quicker and sounder healing and less astigmation than Smith's much more corneal incision. At first sight it might appear that the

[1] *Ophth. Rev.*, August, 1906.

amount of pressure needed to express the lens would be injurious to the eye, but in eight of Maynard's cases "the attempt at expression was abandoned, as the amount of pressure required was considered unjustifiable. The attempt did not appear to have injured the eyeball in any way, as all the eyes obtained good vision." He got a much larger percentage of prolapse of vitreous than Smith's cases show, namely 38 per cent., which is about ten times the percentage of the ordinary operation. This is to our mind a very strong argument against the new operation; and the fact that most cases where vitreous was lost got good vision is not conclusive, for sufficient time has not elapsed for later troubles (detached retina, &c.) to develop, and in India it is almost impossible to follow up cases for any length of time. Maynard has abandoned the operation on the following grounds:—

1. Frequent loss of vitreous with its dangers of detached retina, hemorrhage, and increased risk of infection. 2. Prolonged lowering of the tension with haziness of the cornea and poor vision. 3. Delayed union and an irritable condition of the eye. 4. Frequent rupture of the capsule with its bad effect on vision. "In the face of these drawbacks it is impossible to recommend the performance of the operation." It seems probable that Smith's operation will eventually be confined to cases where the lens is over-mature or where the capsule is opaque.

Five cases of **toxic amblyopia** and one of optic neuritis are recorded as due to thyroid feeding. Birch-Hirschfeld and Inouye have experimented on dogs fed on thyroid tabloids. Four dogs were examined ophthalmoscopically and then fed for periods of from three to ten months on doses increasing up to 8 or 10 grammes of thyroid a day. In one instance the result was *nil*, but in the other three, whilst there was no neuritis or retinitis, the discs became pale and the retinal arteries shrunken. The ganglion cells of the retina on pathological examination showed the same degeneration that has been described in the brain and spinal cord by Edwards. These changes in the ganglion cells were not confined to the macular region, though, as in tobacco amblyopia, the visual defect is most conspicuous in the area of the field corresponding to the macula. It would appear that idiosyncrasy plays as important a part in this as in most toxic amblyopias.

Toxic amblyopia is also attributable in some cases to methyl, or wood alcohol. The blindness, according to Buller and Wood,[1] is bilateral, and may occur a few days or hours after the spirit is taken. Contracted field, central scotoma, slight neuro-retinitis, and subsequently optic atrophy are found. The authors would prohibit the sale of all "deodorised" wood spirit.

[1] *Montreal M. J.*, 1904, xxxiii, 29.

Another case of antipyrin amaurosis is recorded by Hotz.[1] The patient, a man aged 33, suffered from severe ocular and facial neuralgia, for which he took twenty-six 5-grain capsules of antipyrin in forty-eight hours. Towards the end of the course he noticed his sight was failing, and after the last dose he was practically blind. Pallor of the disc and smallness of the retinal vessels were the only ophthalmoscopic changes. There was partial recovery in two days, but it was not complete till after more than six weeks.

A case of transient lead amaurosis is recorded by Otto Loewe.[2] For two days there was complete blindness, but in eight weeks there was perfect recovery. There were no ophthalmoscopic changes. About sixty-five cases of lead amaurosis are on record, which may be divided into two classes: (1) amaurosis with fundus changes (neuritis or optic atrophy); (2) the much rarer condition of transient amaurosis with rapid onset, followed usually by complete recovery, with no ophthalmoscopic changes.

The frequent occurrence of **pneumococci, in hypopyon-keratitis and serpiginous ulcer** of the cornea, has led to the conclusion that it is the specific causal agent in these two conditions. Römer in Bavaria thinks that all cases of ulcus serpens are due to pneumococcal infection, and has initiated a scheme by which every medical practitioner in the district is informed of his methods, so that any case of threatened spreading ulcer may be treated by injection with anti-pneumococcic serum without loss of time. The cost of this is partly paid by public funds and partly by private subscription.

CYRIL H. WALKER.

### Reviews of Books.

[1] *Arch. Ophth.*, 1906, xxxv. 160. [2] *Ibid.*, p. 164.

